# Developing counseling skills through pre-recorded videos and role play: a pre- and post-intervention study in a Pakistani medical school

**DOI:** 10.1186/1472-6920-10-7

**Published:** 2010-01-26

**Authors:** Noor F Ahsen, Syeda A Batul, Ahsen N Ahmed, Sardar Z Imam, Humaira Iqbal, Khayyam Shamshair, Hammad Ali

**Affiliations:** 1 Department of Community Health Sciences.FMH College of Medicine & Dentistry, Lahore, Pakistan; 2 Department of Emergency & Medical Education Council, Allama Iqbal Medical College & Jinnah Hospital Lahore, Lahore, Pakistan

## Abstract

**Background:**

Interactive methods like role play, recorded video scenarios and objective structured clinical exam (OSCE) are being regularly used to teach and assess communication skills of medical students in the western world. In developing countries however, they are still in the preliminary phases of execution in most institutes. Our study was conducted in a naïve under resourced setup to assess the impact of such teaching methodologies on the counseling skills of medical students.

**Methods:**

Fifty four 4^th ^year MBBS students were identified to be evaluated for communication skills by trained facilitators in a pre-intervention OSCE. The same group of students was given a demonstration of ideal skill level by means of videos and role playing sessions in addition to real life interaction with patients during hospital and community rotations. A post-intervention evaluation was carried out six months later through OSCE and direct observation through structured checklist (DOS) in hospital and community settings. The combined and individual performance levels of these students were analyzed.

**Results:**

There was a statistically significant difference in the communication skills of students when assessed in the post-intervention OSCE (p = 0.000). Individual post-intervention percentages of study participants displayed improvement as well (n = 45, p = 0.02). No difference was observed between the scores of male and female students when assessed for two specific competencies of antenatal care and breast feeding counseling (p = 0.11). The mean DOS (%) score of 12 randomly selected students was much lower as compared to the post-intervention (%) score but the difference between them was statistically non significant, a result that may have been affected by the small sample size as well as other factors that may come into play in real clinical settings and were not explored in this study (59.41 ± 7.8 against 82.43 ± 22.08, p = 0.88).

**Conclusions:**

Videos and role play in combination with community and clinical exposure are effective modes of teaching counseling skills to medical students. They can be successfully utilized even in a limited resource setup, as demonstrated by our trial.

## Background

Counseling on health promotion, disease prevention and cure is a fundamental part of any medical consultation. It has a direct impact on patient health and an overall impact on the burden of disease in the community. In a third world country like Pakistan where malnutrition, lack of infant immunization and diarrheal diseases are still significant problems [1], it is imperative for doctors to be able to effectively counsel their patients about common primary health issues [2]. This is only possible if the doctor possesses adequate knowledge on the topic and is able to transmit that information to the patients satisfactorily.

There is evidence that good communication translates into increased patient satisfaction and compliance resulting in better outcomes [3,4]. Although traditionally centered on paternalism, healthcare in Pakistan is gradually evolving into a more individual centered model. This change has been a recipient of mixed response from both care-givers as well as seekers in our setup. In a system where physician-patient communication is invariably hampered by factors like illiteracy, ignorance, poverty, hierarchical family structure and male dominance, an immediate switch to complete patient autonomy is not possible. In the transitory phase most doctors are trying to maintain a balance between autonomy and paternalism [5,6].

Meanwhile medical educators in the developed countries are striving to incorporate communication skills training in the undergraduate and postgraduate curricula [7,8]. Recently, there has been a change in many institutions from the traditional didactic methods towards more interactive methods like role-playing and screening of video-recorded doctor-patient interactions to the students [4,7-9]. The perceived benefits of these methodologies are based on the work of Kolb & Fry who described four learning environments in their theory of experiential learning [10]. They proposed that learning is greatly facilitated when all four 'learning environments' are simultaneously employed: affectively-oriented (feeling), symbolically-oriented (thinking), perceptually-oriented (watching) and behaviorally-oriented (doing). Role-playing and video-recorded scenarios, when combined with actual practice on patients, include all four and are thus currently in vogue for communication skills training [11].

While many schools in the West have introduced formal communication skills training, such a trend has not yet picked up in Pakistan. This study was carried out keeping in mind the need to introduce counseling skills training using interactive methods in Pakistan, followed by its practical application through student-patient encounters. Finally, the objective assessment of the impact of such curricular changes was also addressed in this study.

## Methods

The study was carried out at FMH College of Medicine and Dentistry, a private medical college in Lahore, Pakistan. The college admits an average of 75 students in every class and follows the typical five year curriculum as determined by the Pakistan Medical and Dental Council (PMDC), and the University of Health Sciences (UHS). The first two years are devoted entirely to Basic Health Sciences while clinical rotations are part of the curriculum for the next three years. This study involved fourth year medical students during the session 2006-07.

Before the intervention, students had not received any formal training in communication skills in the medical college. The curriculum did not provide a designated slot for a rotation in Community Medicine as well. Our intervention included a two-week rotation with the Community Medicine Department and four video-recorded scenarios depicting an ideal doctor-patient encounter. Four instructors of the department were trained to counsel for vaccination of infants, diarrhea management, breast feeding and antenatal care. The same instructors were also trained to act as patients. The instructors alternated between acting as patients and physicians to produce videos on these four topics of counseling, each lasting about 15 minutes. The same facilitators also observed and evaluated the students later on, in test as well as real clinical settings.

The videos covered the essential elements of physician-patient communication including the ways to open a consultation, use of open and close ended questions, clarifications, facilitation, reflection and assessment of patient understanding [12]. The videos were prepared completely in *Urdu *(the national language of Pakistan) since the local community in Pakistan mostly does not understand English. To the best of our knowledge, these are the first such video scenarios recorded in *Urdu *for the undergraduate level. All videos were reviewed by a family physician and a psychologist for adequacy of content.

**Figure 1:**
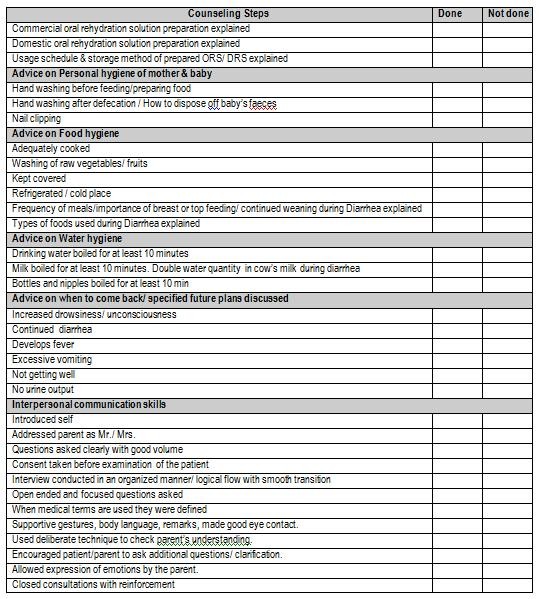
**Checklist used for evaluation of anti diarrhea counseling**.

Students inclusive in the study were evaluated for communication and interpersonal skills (CIS), pre- and post intervention based on a structured checklist. The assessment checklists had been prepared using the Education Commission for Foreign Medical Graduates (ECFMG) scoring criteria for interpersonal skills in clinical skills examination and the Robert Wood Johnson College of Medicine, New Jersey assessment list for OSCEs (Objective Structured Clinical Examinations) in consultation with a family physician and a psychologist [13,14]. All contained a common section on communication skills in addition to a variable number of specific clinical counseling points that the students were supposed to address. One mark was awarded for correctly performing each item on the checklist. There were only two categories, 'done' and 'not done', for each item (Figure 1) To minimize inter-rater disparity each instructor was given one specific category to grade, before and after intervention as well as in real clinical setting for one set of rotation. Additionally, the percentage of items checked by an assessor in different categories was compared periodically. The study question was not revealed to the observers to ensure unbiased assessment.

The checklists used had been pretested on students passing through fourth year in the previous year, to ascertain the feasibility of the study design. This group of students on an average scored similar to the pre-intervention group of the study. They were subsequently utilized in the actual study without any change.

Each participant of the trial underwent an initial assessment at the beginning of the two week rotation in the four selected categories through role playing by trained instructors. Students were made to practice these counseling skills on real patients encountered during their visits to community clinics. These encounters were observed by the same facilitators using guidelines similar to the items inclusive in the assessment checklists. On-the-spot feedback was provided to the students. The post-intervention OSCE was repeated after six months and the results of the two were compared in order to objectively document the effect of the intervention provided [15]. Moreover, one actual real-time student-patient interaction in a clinical or community setting was randomly selected out of the post intervention group for each counseling topic and graded with the same checklist that had been used earlier in the OSCEs (Direct Observation through structured checklist DOS).

Data was entered and analyzed using SPSS 12.0. Paired t-test was performed with the one-tailed probability set at 0.05.

The project was approved by the Institutional Review Board and Ethical committee of FMH College of Medicine & Dentistry, Lahore. The study was in compliance with the 'Ethical Principles for Medical Research Involving Human Subjects' as laid down in the Helsinki Declaration [16]. Since the rotation was part of their curriculum and approved by the academic council of the college, individual informed consent was not required from every student.

## Results

Fifty-four out of 75 fourth year MBBS students (41 females and 13 males) were selected to complete the exercise in twelve batches. Twenty-one had to be excluded from the study as they failed to meet the selection criteria of being regular students who had been promoted at the beginning of the session after passing the university professional examination.

**Figure 2:**
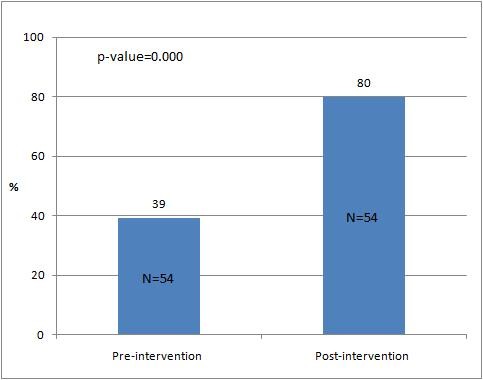
**Mean pre- and post-intervention OSCE score (%) of selected 54 students**.

It was observed that the mean pre-intervention OSCE percentage score for the selected students was 39.33 (SD ± 14.6; range 66). There was a statistically significant difference when the communication skills of the same group were assessed in the post intervention OSCE (80.03 SD ± 13.3; range 64 t-statistics 2.8, p = 0.000, Figure 2). Furthermore, the individual performances of students prior to and following demonstration were reviewed. Majority (n = 45) displayed significant improvement in the post-intervention OSCE percentages (t-statistic: >2, p = 0.02) while nine students failed to achieve any improvement in their communication skills level. The mean scores of male and female students for each competency are shown in Table 1.

**Table 1:** Mean pre- and post intervention OSCE scores of male and female students in four selected competencies

Students distribution according to gender (Total N = 54)	Competencies assessed	Mean % Pre-intervention OSCE score	Standard Deviation (SD)	Mean % Post-intervention OSCE score	Standard Deviation (SD)	P-value
Females N = 41	Breast feeding	49.09	± 26.48	83.63	± 11.81	0.000
	
	Vaccination	46.14	± 20.98	83.19	± 10.70	0.000
	
	Ante-natal care	38.07	± 20.65	86.78	± 14.76	0.000
	
	Anti-diarrhea counseling	34.95	± 23.98	83.00	± 12.28	0.000

						

Males N = 13	Breast feeding	37.46	± 29.82	63.00	± 29.93	0.002
	
	Vaccination	38.92	± 14.52	75.07	± 10.58	0.000
	
	Ante-natal care	29.23	± 17.41	70.84	± 21.61	0.000
	
	Anti-diarrhea counseling	21.15	± 13.89	64.61	± 24.34	0.000

**Figure 3:**
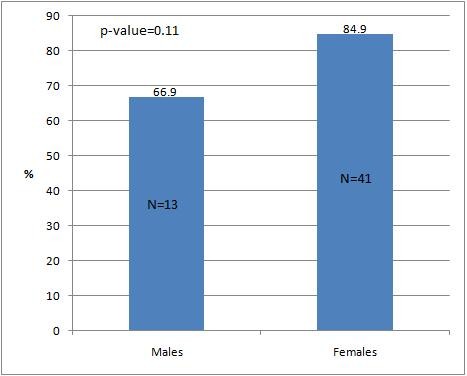
**Comparison of combined mean post-intervention OSCE score (%) for two competencies (ante-natal and breast feeding counseling) for male and female students**.

It was felt necessary to study the effect of gender while dealing with culturally sensitive issues like breast feeding and antenatal care, a potential communication barrier in our country. Although there was a 18 percentage points difference between the post intervention OSCE scores for two combined competencies for male and female students it was not statistically significant (Post-intervention OSCE % scores Males: 66.9 ± 22.8 range 66; Females: 84.9 ± 8.9 range 33.5 t-statistic: 2.8, p = 0.11, Figure 3).

**Figure 4:**
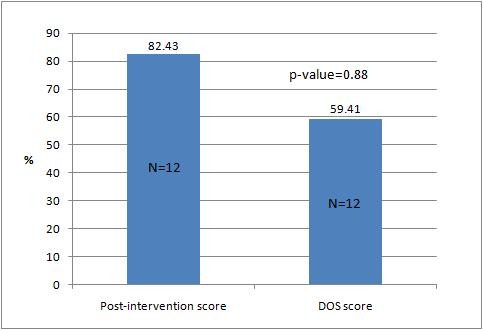
**Comparison of mean percentage post-intervention OSCE score and DOS for selected 12 students**.

An important aspect of this trial was to observe the counseling skills of the students in the post intervention group in a real life setting by a trained facilitator observing their interaction with patients (Direct Observation through Structured Checklist, DOS). Twelve students were randomly selected out of each batch to be monitored. The mean DOS score of all 12 was much lower as compared to the post-intervention % score (59.41 ± 7.8 range: 27.75 against 82.43 ± 22.08 range: 64, p = 0.88; Tables 2 and 3; Figure 4).

**Table 2:** Comparison of mean post-intervention OSCE and DOS scores of selected students in each competency

Competencies assessed	Mean % Post-intervention OSCE score	Standard Deviation (SD)	Mean % DOS score	Standard Deviation (SD)	P-value
Breast feeding	84.91	± 8.44	56.75	± 29.77	0.002

Vaccination	80.91	± 9.85	49.50	± 33.06	0.004

Ante-natal care	80.58	± 14.19	66.20	± 25.07	0.045

Anti-diarrhea counseling	83.33	± 11.61	65.16	± 18.42	0.001

Number of students = 12

**Table 3:** Mean post-intervention OSCE and DOS scores of male and female students in selected competencies

Students distribution according to gender (Total N = 12)	Competencies assessed	Mean % Post-intervention OSCE score	Standard Deviation (SD)	Mean % DOS score	Standard Deviation (SD)	P-value
Males N = 2	Breast feeding	78.00	± 5.65	29.50	± 41.71	0.15
	
	Vaccination	74.00	± 0.00	42.50	± 50.20	0.51
	
	Ante-natal care	87.00	± 2.82	38.50	± 27.57	0.04
	
	Anti-diarrhea counseling	86.50	± 7.77	73.00	± 5.65	0.09

						

Females N = 10	Breast feeding	86.30	± 8.42	62.20	± 26.30	0.12
	
	Vaccination	82.30	± 10.29	50.90	± 32.30	0.16
	
	Ante-natal care	79.30	± 15.31	71.80	± 21.87	0.38
	
	Anti-diarrhea counseling	82.70	± 12.47	63.60	± 19.87	0.01

## Discussion

Medical education in Pakistan requires a mandatory upgrading to the new methods of teaching, particularly in areas less addressed in the past. It is unanimously agreed amongst members of international graduate medical education and physician licensing boards that good counseling skills of health care providers will not only result in high-quality care provision through accurate flow of information between healthcare individuals, teams and patients but increased physician and patient satisfaction as well [17].

A variety of teaching and evaluation tools, are being developed to effectively incorporate CIS into physicians training and standardized assessments [18]. While *simulated patient *(SP) based evaluation is being widely implemented globally as a method of CIS assessment in regular and high stake medical examinations, most medical institutes in this part of the world do not have the finances and man-power to execute it [17,19,20]. It is due to this missing link in our medical education that students face difficulties in foreign licensing exams. Studies have indicated that international medical graduates (IMGs) are more likely to feel pressed for time in clinical skills examination in comparison to the US graduates [21]. Furthermore, about 17% of the IMGs fail the United States Medical Licensing Exam (USMLE) Step 2 Clinical Skills (CS) component mostly due to inadequate interpersonal skills as opposed to only 4% US graduates [22].

Lack of resources to execute performance-based assessment may be one of the reasons that some medical institutes in Pakistan have not taken this initiative. Prerecorded videotapes and objective assessment through role playing by trained facilitators are simple and cost effective alternatives. In addition, the successful incorporation of OSCE in the undergraduate medical curriculum by the faculty of National University at Cuyo, Mendoza, Argentina, despite the deficiency of technological and staffing resources, demonstrates that initiatives can be taken in resource-limited environments as well [23]. Our trial in similar circumstances showed a very encouraging outcome albeit with certain limitations.

Due to the ethical concern of depriving students of an exercise that had been officially made part of the curriculum it was not possible to establish a true control group. The selected students, before intervention served as their own control. Being an independently planned and funded study, the ideal randomized control multi-centric trial could not be executed. The findings from this study are invaluable still, and can serve as the basis of conducting a more elaborate one in the future.

The unsatisfactory performance of students in real patient encounters is probably due to multiple factors that were not explored in this study, the leading ones being: a careless attitude when in a non-testing environment, language barrier (different local dialects), difficulty in adapting to hospital and outpatient departments, unease when dealing with non-familiar people etc. More importantly, the small sample size and limited number of encounters observed per student has also had an impact on the outcome. It is not possible to predict conclusively, the magnitude of the effect our intervention has had on the CIS of the selected students without a more robust experimental study. The next step should be to revise our medical schools curricula to include a regular CIS course and observe its effect on the performance level of students when all other confounding factors have been accounted for.

Based on this study, we suggest that medical schools of the developing world particularly Pakistan, should take initiatives to not only revise their curricula but train the facilitators to improvise teaching methodologies as well. This can be achieved with some effort on the part of the medical education departments and administration and does not necessarily imply the investment of multiple resources. Introduction of formal courses in communication skills and subsequent assessment can serve to build the competence level of the medical students [24]. Significant evidence is available of the utility of such initiatives and their potential contribution to producing more effective health care providers [25].

## Conclusion

Pre-recorded videos and role play are simple, cost effective tools for demonstrating counseling skills to medical students in addition to clinical and community exposure. The exact magnitude of the impact of intervention on the communication skills of students cannot be predicted in view of the limitations. However, it still offers significant evidence towards successful implementation of a formal communication skills development initiative, under resource limited circumstances.

## Competing interests

The authors declare that they have no competing interests.

## Authors' contributions

NFA participated in the design of the study, acquisition, analysis and interpretation of data, manuscript drafting and final approval of the version to be published. SAB participated in the acquisition, analysis and interpretation of data and drafting of manuscript. ANA was involved in the design of the study, analysis and interpretation of data and final approval of the version to be published. SZI participated in the data acquisition, interpretation and manuscript drafting. HI, KS and HA participated in data acquisition. All authors have read and approved of the final manuscript.

## Authors' Information

NFA- *FCPS *(Community Medicine), Fellow Health Professions Education *FHPE *(FAIMER, USA) Professor of Community Health Sciences, FMH College of Medicine and Dentistry, Lahore, Pakistan.

SAB- *MBBS, M.D *Instructor Community Health Sciences, FMH College of Medicine and Dentistry, Lahore, Pakistan

ANA- *FCPS *(Surgery), Additional Director Accident and Emergency Jinnah Hospital, Lahore, Pakistan; Member of the Medical Education Council Allama Iqbal Medical College, Lahore, Pakistan

SZI- *MBBS, M.D *Instructor Community Health Sciences, FMH College of Medicine and Dentistry

HI-*MBBS, M. Phil *(Community Medicine) Senior Instructor Community Health Sciences, FMH College of Medicine and Dentistry, Lahore, Pakistan

KS- *MBBS *Instructor Community Health Sciences, FMH College of Medicine and Dentistry, Lahore, Pakistan

HA- *MBBS *Instructor Community Health Sciences, FMH College of Medicine and Dentistry, Lahore, Pakistan.

## Pre-publication history

The pre-publication history for this paper can be accessed here:

http://www.biomedcentral.com/1472-6920/10/7/prepub
